# Critical analysis of the prescription and evaluation of monoclonal antibodies approved in Germany for the outpatient setting from 2010—2021

**DOI:** 10.1007/s00210-026-05201-3

**Published:** 2026-04-07

**Authors:** Anna Lena Tödter, Roland Seifert

**Affiliations:** Institute of Pharmacology, Hannover Medical School, Carl-Neuberg-Str. 1, 30625 Hannover, Germany

**Keywords:** Targeted drugs, Early benefit assessment, Rational prescribing behavior, Pharmaceutical costs, AVR, Monoclonal antibodies

## Abstract

Monoclonal antibodies (mAbs) are increasingly being launched on the German market and account for a growing share of total drug costs every year. In 2021 alone, eleven monoclonal antibodies were among the 30 best-selling drugs, accounting for 9.91% of total drug costs. In contrast, a considerable additional benefit of the newly introduced monoclonal antibodies is not predominantly demonstrable. Therefore, our objective was to analyze the drug prescriptions and associated costs in comparison to the benefit assessment of the Federal Joint Committee (GBA). This paper analyzes 62 monoclonal antibodies that were approved by the European Medicines Agency in the period from 2010 to 2021 and newly launched on the German market. The data basis is provided by the Drug Prescription Report (AVR) and the Statutory Health Insurance (SHI) Drug Index for outpatients. The SHI index is compiled by the Wissenschaftliches Institut der AOK (WIdO). The GBA provides a further data basis for the assessment of additional benefits. The relevant parameters used for each drug include the number of prescriptions, net costs, defined daily doses (DDD) and costs of the defined daily doses (DDD costs) at the time of the year of approval and as of 2021. There is only a slight correlation between the number of DDDs, prescriptions and net costs of the respective drug and the additional benefit assessed by the GBA. There is a certain correlation between the advertisements in the journal “Oncology Research and Treatment” and the drug benefit. In summary, the high costs of monoclonal antibodies cannot be justified by the additional benefit assessed by the GBA, as no overriding significant additional benefit can be proven for the drugs analyzed. More precise regulation of drugs with little or no additional benefit, as well as regulation of the price of drugs with considerable additional benefit, is crucial to ensure a better balance between costs and benefits.

## Introduction

The net pharmaceutical expenditure of statutory health insurance (SHI) in Germany has been on a strong upward trend for years. In the years from 2016 to 2021 alone, costs rose by 49.7%. This corresponds to net pharmaceutical expenditure of €50.3 billion in 2021, of which the eleven best-selling monoclonal antibodies alone account for €4.98 billion in 2021 (Ludwig et al. 2023).

Despite representing a mere 0.35% of prescriptions in the overall market, monoclonal antibodies account for 8.6% of total costs. These numbers impressively show that a very small proportion of prescriptions is spent on high-cost drugs.

The leading drug group by far is oncologics, of which monoclonal antibodies account for the largest share at € 4,323.9 billion. A correlation between an additional clinical benefit and the high costs is not present for the monoclonal antibodies (Obst and Seifert 2023). Monoclonal antibodies are targeted therapies and are being used more and more frequently in therapy of malignant diseases or immunosuppression, which gives them increasing relevance (Ludwig et al. 2022). Therefore, the goal of our present study was to assess the clinical benefit of monoclonal antibodies in relation to their costs.

## Methods

The analyses in this paper are based on the prescription data from the Drug Prescription Report (AVR) from 2010 to 2022 and the Statutory Health Insurance Drug Index for outpatients. This is compiled by the Scientific Institute of the AOK (WIdO) under the auspices of the AOK Federal Association. Furthermore, the analyses are based on the benefit assessment of the Federal Joint Committee (GBA) and the Federal Committee for Health. In the analysis, only drugs with new active ingredients were taken into account and already known drugs with new combinations of active ingredients or new indications were excluded. Furthermore, biosimilars were excluded in this analysis, because no biosimilars of the monoclonal antibodies analysed were available on the market during the period covered by this analysis. The relevant parameters used in this analysis are the number of prescriptions, the net costs and the defined daily doses (DDD) per drug at the time of approval and are compared with the value in the year 2021.

The prescription data is based on the drug prescriptions that doctors issue for insured persons. These prescriptions are billed by public pharmacies and hospital pharmacies in outpatient care and are paid for by the statutory health insurance (SHI). These data do not include non-prescription medicines that are not reimbursed by statutory health insurance, for example for children. Likewise, private purchases for self-medication, i.e. so-called OTC medicines, and prescriptions issued as private prescriptions are not included. Since 2013, parenteral solutions and cytostatic preparations have also been included in the prescriptions, while since 2018, dispensing and blistering have also been included in the data. These four groups of medicines are specially prepared by pharmacies for individual patients, particularly in the field of cancer and immunotherapy. Finished medicinal products are adapted to individual dosages, which are then provided with corresponding billing information by the pharmacies. Since 2019, all data from hospital pharmacies in the context of outpatient care have also been fully recorded (Schröder et al. 2021).

This analysis excludes drugs that are used for inpatients in hospitals. The reason for the exclusion is that in Germany the hospital billings are usually based on flat rates, so-called “diagnosis-related groups” (DRG). Consequently, pharmaceuticals are not invoiced separately but rather incorporated into the overall flat-rate per case structure. In consequence, there is an absence of accurate, puclicly available projections of the precise number of DDDs, prescriptions and costs of drugs in hospitals, because such data is generally included in the DRGs or, where relevant, negotiated individually with manufacturers by each hospital (GKV Spitzenverband 2026; Reimbursement Institute 2026).

The net costs of the respective drugs are based on the actual costs incurred by the statutory health insurer and its insured persons. They are composed of the gross turnover, which reflects the cost of a drug according to the valid pharmacy retail price, minus the fixed manufacturer and pharmacy discounts. The net costs also include the insured person’s own contribution, which in Germany corresponds to a percentage co-payment of 10%, with a minimum of €5 and a maximum of €10, as well as a value added tax (VAT) (Schröder et al. 2021).

The prescriptions per drug correspond to the number of prescribed packs, irrespective of the number of units (e.g. tablets, syringes) contained in the pack (Schröder et al. 2021). The defined daily dose (DDD) is defined as the assumed maintenance dose used on average per day for a medicine in adults. The DDD does not reflect the recommended or actual prescribed dose, but serves as a unit of measurement and is independent of price, currency, package size and potency. If the DDD refers to a body weight, a person of 70 kg is assumed (WHO Collaborating Centre for Drug Statistics Methodology et al. 2018; Wissenschaftliches Institut der AOK 2022). The advantage of the DDD is the improved comparability between the dosages of the various drugs, as consumption is measured on the basis of a fixed quantity of active ingredient and therefore changes in pack sizes or the use of other dosage strengths do not distort the measured consumption. The DDD is an international measure defined by the WHO. The DDD generally refers to the information for outpatient dosing. If a drug has both an initial dose and a maintenance dose, the DDD generally refers to the maintenance dose. The DDD costs are the average net costs for a prescribed daily dose of a specific drug or group of drugs. These costs are calculated as an average of all prescribed variants and weighted according to the respective prescribed package quantity. The costs per DDD in euros reflect the average treatment costs of a drug on one day (Schröder et al. 2021). Deviations from the values from previous publications may result from updated extrapolations of previous prescription years. A total of 62 monoclonal antibodies are analyzed, which can be assigned to 11 different superordinate indication areas. Table 1 provides an overview of the drugs analyzed.

**Table 1:** Overview of the drugs analyzed from 2010 - 2021 and their indication areas. O, orphan drug status

	Pharmaceuticals	Year of registration	DDD/DDD costs in thousand. After full year of registration	DDD/DDD costs in thousand 2021	Initial Indication at time of approval
1	Tralokinumab (Adtralza)	2021	Not applicable	Not applicable	Moderate-severe atopic dermatitis
2	Bimekizumab (Bimzelx)	2021	Not applicable	Not applicable	Moderate-severe plaque psoriasis
3	Ofatumumab (Kesimpta)	2021	Not applicable	Not applicable	Active relapsing–remitting multiple sclerosis
4	Satralizumab (Enspryng) (O)	2021	Not applicable	Not applicable	Neuromyelitis optica spectrum disorder
5	Dostarlimab (Jemperli)	2021	Not applicable	Not applicable	Recurrent or advanced endometrial cancer with dMMR and MSI-H, during or prior platinum-based therapy
6	Tafasitamab (Minjuvi) (O)	2021	Not applicable	Not applicable	In combination with Lenalidomide followed by monotherapy, relapsed or refractory DLBCL, ASZT is not an option
7	Isatuximab (Sarclisa)	2021	Not applicable	Not applicable	In combination with Pomalidomide and Dexamethasone, relapsed and refractory multiple myeloma, with at least two previous therapies with Lenalidomide and proteasome inhibitor
8	Brolucizumab (Beovu)	2020	990.7/36.49	990.7/36.49	Neovascular age-related macular degeneration
9	Ibalizumab (Trogarzo)	2020	0.7/333.80	0.7/333.80	In combination with multidrug-resistant HIV-1 infection
10	Crizanlizumab (Adakveo) (O)	2020	18.3/219.15	18.3/219.15	Prevention of recurrent vaso-occlusive crises in patients with sickle cell disease
11	Romosozumab (Evenity)	2020	290/23.12	290/23.12	Manifest osteoporosis in postmenopausal women with significantly increased fracture risk
12	Belantamab mafodotin (Blenrep) (O)	2020	35.5/647.94	35.5/647.94	Multiple myeloma, received at least four prior therapies
13	Polatuzumab (Vedotin) (Polivy)	2020	40.9/529.10	40.9/529.10	Recurrent or refractory DLBCL, in combination with Bendamustine and Rituximab
14	Mogamulizumab (Poteligeo)	2020	25/383.41	25/383.41	Mycosis fungoides or Sezary syndrome with at least one previous systemic therapy
15	Risankizumab (Skyrizi)	2019	973.5/57.02	1776.6/54.65	Moderate to severe plaque psoariasis
16	Ravulizumab (Ultomiris)	2019	125.8/1008.38	197.6/1003.44	Paroxymal nocturnal hemoglobinuria
17	Galcanezumab (Emgality)	2019	687.4/15.57	1082.7/15.37	Migraine prophylaxis for at least 4 migraine days per month
18	Fremanezumab (Ajovy)	2019	762.7/15.27	1712.5/14.64	Migraine prophylaxis for at least 4 migraine days per month
19	Cemiplimab (Libtayo)	2019	92.6/332.45	144.9/205.45	Metastasized or locally advanced cutaneous squamous cell carcinoma
20	Lanadelumab (Takhzyro) (O)	2019	47.8/1053.71	61.4/1052.37	Prophylaxis of attacks of hereditary angioedema
21	Tildrakizumab (Ilumetri)	2018	393.7/49.66	1533.5/38.28	Moderate to severe plaque psoariasis
22	Bezlotoxumab (Zinplava)	2018	< 0.1/2128.52	< 0.1/1905.69	Prevention of recurrence of Clostridium difficile infection
23	Benralizumab (Fasenra)	2018	785.2/45.60	1325/44.05	Severe eosinophilic asthma, add-on maintenance therapy
24	Caplacizumab (Cablivi) (O)	2018	1.2/5181.06	2.8/4382.33	Acquired thrombotic thrombocytopenic purpura
25	Emicizumab (Hemlibra)	2018	29.8/1419.15	127.7/1062.53	Routine prophylaxis for hemophilia A with factor VIII inhibitors
26	Erenumab (Aimovig)	2018	2473.5/19.10	5387.2/10.53	Migraine prophylaxis for at least 4 migraine days per month
27	Ocrelizumab (Ocrevus)	2018	2065.4/75.02	3847.1/67.43	Relapsing multiple sclerosis
28	Durvalumab (Imfinzi)	2018	190.7/262.25	417.1/223.85	NSCLC with PD-L1 expression
29	Gemtuzumab/ozogamicin (Mylotarg) (O)	2018	< 0.1/3353.71	< 0.1/3394.28	CD33-positive AML, in combination with Daunorubicin and Cytarabine, not pretreated
30	Burosumab (Crysvita) (O)	2018	48.8/744.89	79.7/779.48	X-linked hypophosphatemia with radiographic evidence of bone disease
31	Guselkumab (Tremfya)	2017	540.6/70.45	3118.4/53.95	Moderate to severe plaque psoariasis
32	Brodalumab (Kyntheum)	2017	315.6/57.04	749.9/49.49	Moderate to severe plaque psoariasis
33	Ixekizumab (Taltz)	2017	890.1/56.89	2913.7/49.69	Moderate to severe plaque psoariasis
34	Dupilumab (Dupixent)	2017	895.4/58.83	5730.4/50.48	Moderate to severe atopic dermatitis
35	Reslizumab (Cinqaero)	2017	67.9/39.88	23.4/39.73	Additional therapy for severe eosinophilic asthma
36	Sarilumab (Kevzara)	2017	237.4/53.99	705.4/49.54	Moderate to severe rheumatoid arthritis after previous therapy with disease-modifying antirheumatic drugs, in combination with Methotrexate or monotherapy
37	Avelumab (Bavencio) (O)	2017	35.4/266.96	160.9/230.08	Monotherapy of metastatic Merkel cell carcinoma
38	Inotuzumab/ozogamicin (Besponsa) (O)	2017	3.8/1412.06	3.9/1131	Relapsed or refractory CD22-positive B-precursor ALL
39	Atezolizumab (Tecentriq)	2017	197/268.46	1233.2/182.1	Locally advanced or metastatic urothelial carcinoma after prior platinum-containing chemotherapy
40	Mepolizumab (Nucala)	2016	627/52.88	1301.9/44.8	Severe refractory eosinophilic asthma
41	Elotuzumab (Empliciti)	2016	102.7/207.67	217.9/180.9	Multiple myeloma, in combination with Lenalidomide and Dexamethasone, at least one previous therapy
42	Daratumumab (Darzalex) (O)	2016	444.3/228.45	2775.4/187.09	Relapsed and refractory multiple myeloma, already pre-treated
43	Necitumumab (Portazza)	2016	3.7/124.53	< 0.1/61.56	Locally advanced or metastatic, EGFR-expressing, squamous non-small cell lung cancer, in combination with Gemcitabine and Cisplatin
44	Olaratumab (Lartruvo) (O)	2016	39.3/355.49	No longer on the market	Advanced soft tissue sarcoma, in combination with Doxorubicin
45	Dinutuximab(Unituxin) (O)	2016	Not assigned by the WHO	Not assigned by the WHO	High-risk neuroblastoma in patients aged 1—17 years
46	Idarucizumab (Praxbind)	2016	Not assigned by the WHO	Not assigned by the WHO	Antidote for Dabigatran
47	Secukinumab (Cosentyx)	2015	2536.7/59.79	6411/54.79	Moderate to severe plaque psoariasis
48	Blinatumomab (Blincyto) (O)	2015	3.6/1421.21	5.3/1071.09	Philadelphia chromosome neg, recurrent or refractory B-precursor ALL
49	Nivolumab (Opdivo)	2015	956.6/240.54	2252.4/209.49	Advanced melanoma, metastatic or locally advanced NSCLC
50	Ramucirumab (Cyramza) (O)	2015	180.7/237.55	377.2/160.57	Advanced adenocarcinoma of the stomach, after previous chemotherapy containing platinum or fluoropyrimidine
51	Pembrolizumab (Keytruda)	2015	272.7/283.25	4264.9/264.9	Non-resectable or metastatic advanced melanoma
52	Alirocumab (Praluent)	2015	663.4/17.27	702.1/10.66	Hypercholesterolemia and mixed dyslipidemia
53	Evolocumab (Repatha)	2015	353.8/24.34	6996.5/15.88	Hypercholesterolemia and mixed dyslipidemia
54	Siltuximab (Sylvant) (O)	2014	0.2/276.4	9.6/214.58	Multicentric Castleman’s disease
55	Obinutuzumab (Gazyvaro) (O)	2014	1.6/209.86	316.4/165.58	CLL in combination with Chlorambucil
56	Trastuzumab emtansine (Kadcyla)	2014	11/228.86	647.6/214.7	HER2-positive, inoperable locally advanced or metastatic breast cancer
57	Vedolizumab (Entyvio)	2014	960.7/60.49	7929.1/37.68	Moderate to severe active ulcerative colitis or moderate to severe Crohn’s disease
58	Pertuzumab (Perjeta)	2013	7.5/157.14	1881.4/123.69	HER2 + metastatic or locally recurrent, unresectable breast cancer, in combination with Trastuzumab and Docetaxel
59	Ipilimumab (Yervoy)	2011	8.0/555.29	237.0/433.77	Advanced, non-resectable or metastasized melanoma
60	Belimumab (Benlysta)	2011	54.7/50.92	612.6/33.97	Additional therapy for autoantibody-positive SLE
61	Denosumab (Prolia)	2010	9004.8/1.84	61,458.8/3.54	Postmenopausal osteoporosis
62	Ofatumumab (Arzerra) (O)	2010	0.5/353.53	No longer on the market	CLL

### Assessment of the added benefit by the GBA

The benefit assessments of the Federal Joint Committee (GBA) are used to analyze the additional benefit of the respective drugs. The GBA defines the additional benefit of a medicinal product as a benefit that is qualitatively or quantitatively greater than the benefit of the appropriate comparator therapy (Gemeinsamer Bundesausschuss 2009). The extent of the additional benefit and the therapeutic significance can be quantified in different categories. These categories differentiate between a considerable, a substantial, a minor, a lesser and a non-quantifiable additional benefit, as well as no additional benefit. The different categories relate primarily to patient-relevant endpoints such as morbidity, mortality and quality of life and thus the strength of the drug’s influence on these endpoints. A non-quantifiable additional benefit is assigned as an assessment according to the GBA if the scientific data basis does not allow quantification. This does not mean that there is no additional benefit, but that there may be an additional benefit that cannot be quantified due to the data situation. (Gemeinsamer Bundesausschuss n.d.). Furthermore, the additional benefit assessment by the GBA for the monoclonal antibodies with an oncological indication are compared with the drug evaluation of the European Society for Hematology and Oncology (ESMO), in regard of similarities and differences. The ESMO-Magnitude of Clinical Benefit Scale (ESMO-MCBS) is a dynamic tool designed to identify cancer treatments that significantly enhance either patient survival or quality of life, setting them apart from therapies with only modest or marginal benefits. It assigns benefit scores to cancer medicines approved by the EMA and the FDA (since January 2020), based on the outcomes of positive randomized clinical trials. The assessment takes into account various factors such as overall survival, progression-free survival, disease-free survival, hazard ratio, response rate, quality of life, disease prognosis, and side effects. Drugs that are classified as ESMO-MCBS scores of A and B for curative therapies and 4 and 5 for non-curative therapies are rated with as a substantial benefit and should be emphasized for faster evaluation of their value and cost-effectiveness. Although a high ESMO-MCBS score does not necessarily imply a high value, since this also depends on the price (European Society for Medical Oncology 2025). Finally a further assessment of the added benefit, evaluations and statements from the German Medical Commission (AKDÄ) in the case of Vedolizumab and relevant studies from the Pubmed register were also used.

## Results

The following section describes the results of the prescriptions, net costs and DDD. In the following analysis, the seven monoclonal antibodies that were newly launched on the market in 2021 (Table 1) are excluded from the analysis, as no reference values regarding the development of prescriptions, net costs or DDD are available at the time of the evaluation. Therefore, 55 monoclonal antibodies are referred to below.

### Net costs (*n* = 55)

**Fig. 1:**
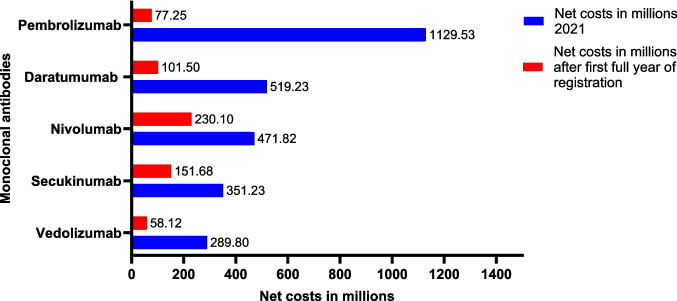
TOP 5 monoclonal antibodies in the net cost category 2021

For 49 of the 55 monoclonal antibodies, an increase in net costs can be demonstrated in the period from initial approval to 2021. For four monoclonal antibodies (Necitumumab, Reslizumab, Gemtuzumab Ozogamicin, Polatuzumab Vedotin), the net costs are decreasing compared to the time of initial launch and 2021. No comparative data can be found for two monoclonal antibodies (Dinutuximab, Olaratumab). Olaratumumab was initially launched in 2016 with the indication for advanced or metastatic soft tissue sarcoma in combination with Doxorubicin. The approval of Olaratumab was revoked in 2019, due to ineffectiveness (Ärzte Zeitung 2019). Dinutuximab was also launched in 2016 with the indication for high-risk neuroblastoma in patients aged 1–17 years. The company has withdrawn the drug in march 2017 from the European market (Pharmazeutische Zeitung 2020). Pembrolizumab has by far the highest net costs in 2021 at € 1.13 billion. With a difference of around € 610 million, Daratumumab follows with net costs of € 519.23 million, followed by Nivolumab with € 471.8 million, Secukinumab with € 351.23 million and Vedolizumab with € 298.8 million (Fig. 1).

Necitumomab has the lowest net costs at € 2.9 thousand, followed by Idarucizumab at € 3.6 thousand, Gemtuzumab Ozogamicin at € 42 thousand, Bezlotoxumab at € 77.9 thousand and Ibalizumab at € 236.4 thousand (Wissenschaftliches Institut der AOK n.d.).

### Prescriptions (*n* = 55)

**Fig. 2:**
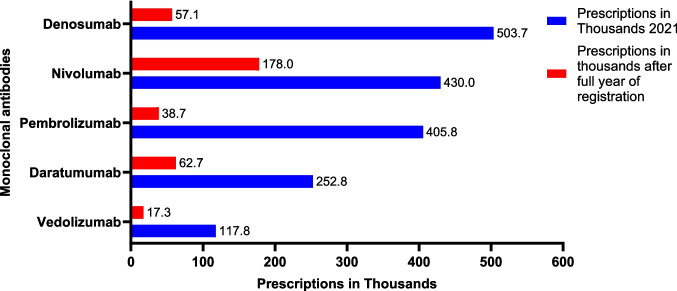
TOP 5 monoclonal antibodies in the Prescriptions 2021 category

The number of prescriptions for the 55 monoclonal antibodies shows an increase for 48 (87.27%) of the 55 drugs in the comparison between the year of approval and 2021. There is a decrease in the number of prescriptions for two drugs (Reslizumab, Necitumumab) and no comparative data can be found for five drugs (Bezlotoxumab, Gemtuzumab Ozogamicin, Dinutuximab, Idarucizumab, Olaratumab). Denosumab is at the top of the list of prescriptions in 2021 with 503.7 thousand prescriptions, followed by Nivolumab with 430 thousand prescriptions, Pembrolizumab with 405.8 thousand prescriptions, Daratumumab with 252.8 thousand prescriptions and Vedolizumab with 117.8 thousand prescriptions (Fig. 2). The lowest prescriptions in 2021 are for Necitumumab, Bezlotoxumab, Idarucizumab and Gemtuzumab Ozogamicin, each with < 0.1 thousand prescriptions, and Ibalizumab with 0.1 thousand prescriptions (Wissenschaftliches Institut der AOK).

#### DDD

**Fig. 3:**
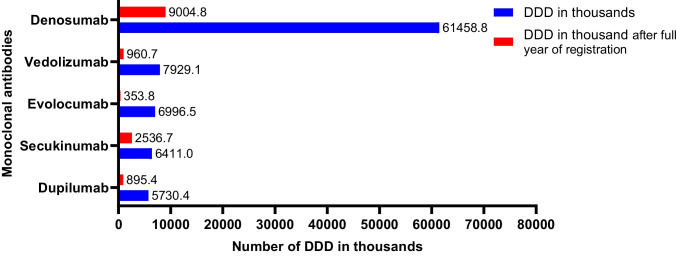
TOP 5 monoclonal antibodies in the DDD category 2021

In the DDD category, there has been an increase in the number of DDDs since the approval year and 2021 for 47 of the 55 monoclonal antibodies. The number of DDDs has decreased for the three monoclonal antibodies Necitumomab, Reslizumab and Polatuzumab Vedotin. No comparative data could be found for five monoclonal antibodies (Olaratumab, Dinutuximab, Idarucizumab, Gemtuzumab Ozogamicin, Bezlotoxumab). Denosumab leads by far in the category of DDDs in 2021 with 61.5 million DDDs (Fig. 3). It is followed by Vedolizumab with 7.9 million, Evolocumab with 7 million, Secukinumab with 6.4 million and Dupilumab with 5.7 million DDDs (Ludwig et al. 2022; Wissenschaftliches Institut der AOK n.d.). Necitumomab, Idarucizumab, Gemtuzumab Ozogamicin, Bezlotoxumab and Ibalizumab also have the fewest DDDs in the DDD category.

As not all monoclonal antibodies from the TOP 5 net costs category are also represented in the TOP 5 prescriptions or TOP 5 DDD category, the relevant mAbs are grouped together in this analysis to form the TOP 8. This concerns Pembrolizumab, Daratumumab, Nivolumab, Secukinumab, Dupilumab, Vedolizumab, Evolocumab. Pembrolizumab and Nivolumab are PD-1 inhibitors that are used in advanced melanoma (European Medicines Agency 2025; Fessas et al. 2017). Daratumumab binds to CD38 and thus activates immune cells and the complement system and is used in multiple myeloma. The IL17A inhibitor Secukinumab is used for moderate to severe plaque psoriasis and the IL-4/14 receptor inhibitor for moderate to severe atopic dermatitis. Vedolizumab is an integrin antagonist used in ulcerative colitis and Crohn’s disease. Evolocumab is a PCSK9 inhibitor used in hypercholisterinemia and mixed dyslipidemia and Denosumab is a RANKL inhibitor with the indication of postmenopausal osteoporosis (EMA 2022). Necitumomab, Idarucizumab, Gemtuzumab Ozogamicin, Bezlotoxumab and Ibalizumab all have the lowest combined share in the three categories of prescriptions, net costs and DDDs and are referred to below as FLOP 5 (Tables 2 and 3). Necitumumab is an EGFR inhibitor used in metastatic NSCLC. Idarucizumab is a Dabigatran antagonist and Gemtuzumab Ozogamicin is used as a CD33-targeted cytostatic inhibitor. Bezlotoxumab neutralizes toxin B of Clostridium Dificile and Ibalizumab binds to CD4-positive T cells and blocks the binding of the HIV-1 virus (European Medicines Agency n.d.).

**Table 2:** TOP 8 monoclonal antibodies from 2010—2021

Drug substance	Additional benefit G-BA	Indication area
Pembrolizumab	Considerable	Oncological diseases
Daratumumab	Considerable	Oncological diseases
Nivolumab	Considerable	Oncological diseases
Secukinumab	Considerable	Skin diseases
Dupilumab	Considerable	Skin diseases
Vedolizumab	Not documented	Diseases of the digestive system
Evolocumab	Not documented	Metabolic diseases
Denosumab	Proceedings discontinued	Diseases of the musculoskeletal system

**Table 3:** FLOP 5 of monoclonal antibodies from 2010—2021

Drug substance	Benefit assessment G-BA	Indication area
Idarucizumab	No G-BA assessment	Antidote Dabigatran
Bezlotoxumab	Low	Infectious diseases
Ibalizumab	Not documented	Infectious diseases
Necitumumab	Not documented	Oncological diseases
Gemtuzumab ozogamicin (O)	Not quantifiable	Oncological diseases

### Development of monoclonal antibodies

**Fig. 4:**
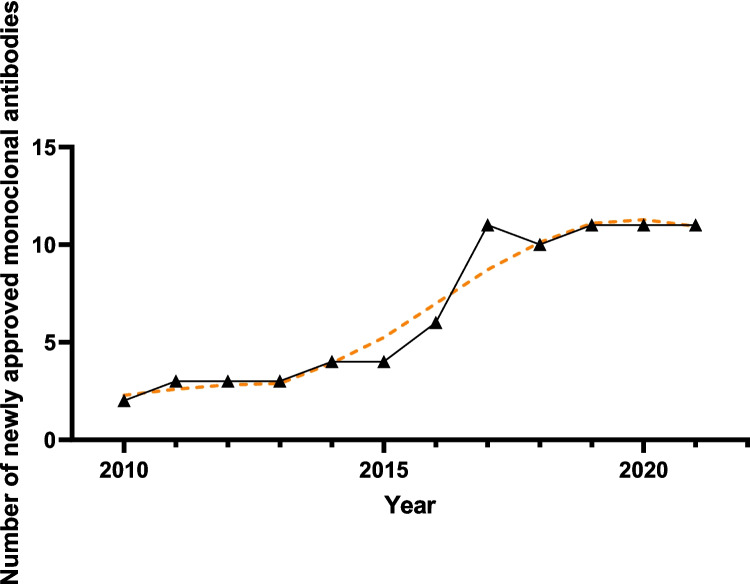
Number of newly approved monoclonal antibodies per year from 2010–2021. The orange line represents a non-linear regression analysis of the approvals. (*n* = 62)

In the period from 2010 to 2021, a total of 220 new drugs were approved, 62 of which were monoclonal antibodies. This corresponds to a percentage share of 28.2%. While monoclonal antibodies only accounted for a small share of 8.7% of newly approved medicinal products between 2010 and 2014, the number doubled to 18.9% in 2015 and rose to 27% by 2018 (Fig. 4). The share declines slightly from 2019 and amounts to 18.4% in 2021 (Ludwig et al. 2022).

**Fig. 5:**
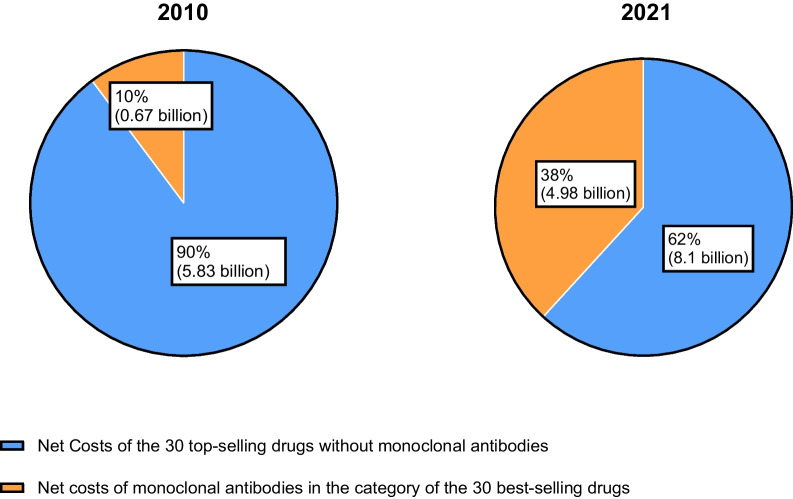
Share of the net costs of mAbs in the 30 top-selling pharmaceuticals in 2010 and 2021

In recent years, there has not only been an increase in the number of newly approved monoclonal antibodies, but also an increase in the number of mAbs, which are among the 30 best-selling drugs (Fig. 5). While the net costs of monoclonal antibodies accounted for 10.19% of the net costs of the 30 top-selling pharmaceuticals in 2010, this figure increased to 18,28% in 2015 and then to 38.21% in 2021.  This means that in 2021, 11 monoclonal antibodies accounted alone for 9.91% of total drug costs.

The oncology drug group has been shown to incur significantly higher net costs than other drug groups (Table 4). Monoclonal antibodies account for the largest share of this, with net costs amounting to €4.3 billion. The net costs of oncology drugs are approximately 3.5 times higher than those of antidiabetic drugs and almost 6 times higher than the net costs of antipsychotic drugs. In contrast, the number of prescriptions for oncology drugs corresponds to only a quarter of the number of antidiabetic drugs and only 16% of the number of antipsychotic drugs. Similarly, the number of DDDs for oncology drugs is only approximately 11% of the number of antidiabetic and antipsychotic drugs *(*Ludwig et al. 2022*)*.

**Table 4:** Comparison of different drug groups and their net costs, number of prescriptions and number of DDD in 2021. Data taken from Ludwig et al. (2022)

Drug Group	Net cost in million €	Prescriptions in million	DDD in million
Oncologics	10,625.56	8.42	272.77
Antidiabetics	3,051.86	32.89	2,504.39
Analgetics	1,820.83	52.9	743.31
Antipsychotics	1,790.79	50.66	2,507.17

**Fig. 6:**
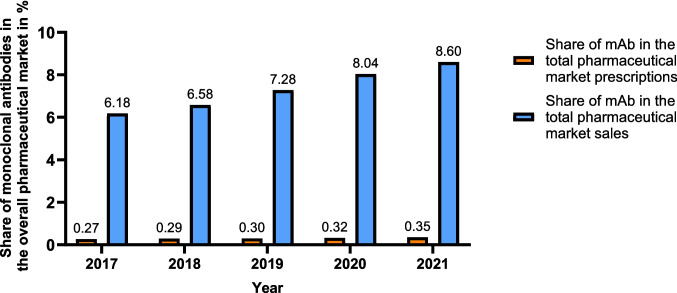
Share of mAbs in the total pharmaceutical market from 2017—2021

In 2017, monoclonal antibodies accounted for 0.27% of prescriptions in the total pharmaceutical market, while the net cost of monoclonal antibodies accounted for 6.18% of the total pharmaceutical market in 2017 (Fig. 6). This means that the net costs of monoclonal antibodies account for a 23-fold higher share of the total pharmaceutical market than the number of monoclonal prescriptions in the total number of prescriptions.

In 2021, the share of prescriptions for monoclonal antibodies amounts to 0.35% of the total pharmaceutical market and the share of net costs to 8.60%. This corresponds to an increase of 0.08% in prescriptions and an increase of 2.42% in net costs, which equates to an increase of €1.9 billion. (Ludwig et al. 2021; Schwabe et al. 2019; Schwabe et al. 2018; Schwabe et al. 2017; Schwabe and Ludwig 2020). 

### Indication areas

**Fig. 7:**
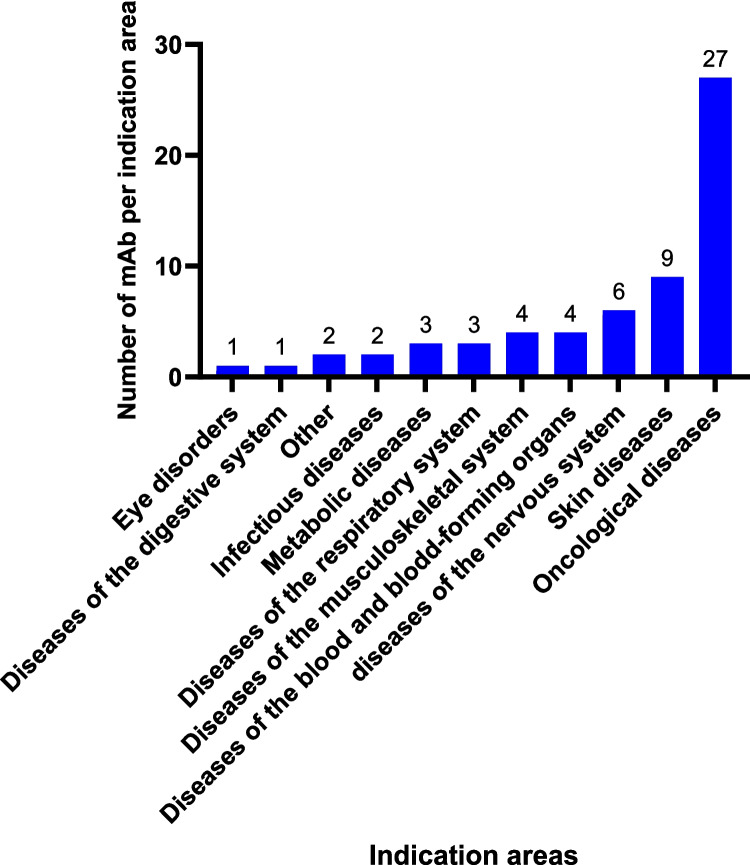
Number of mAbs per initial indication area at time of approval (*n* = 62)

The largest overarching indication area for monoclonal antibodies between 2010 and 2021 is by far oncological diseases (Fig. 7). This is followed at some distance by skin diseases and diseases of the nervous system. Diseases of the blood and blood-forming organs and diseases of the musculoskeletal system, as well as diseases of the respiratory system and metabolic diseases follow with a smaller share. Infectious diseases, diseases of the digestive system, eye diseases and other indications account for the smallest two shares. Figure 7 refers to the initial indication of a newly approved monoclonal antobody.

### Additional benefit assessment by the GBA

**Fig. 8:**
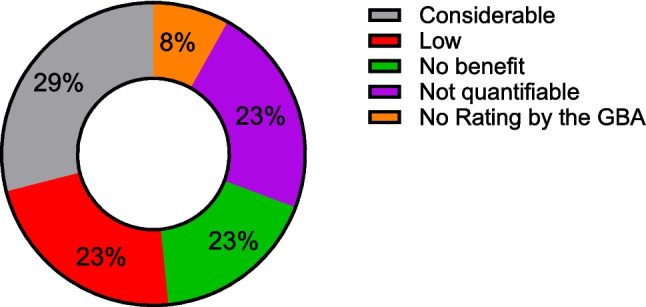
GBA assessment of the added benefit 2011–2021 (*n* = 62). Values rounded; discrepancies due to rounding

In the assessment of the added benefit by the JCC, a considerable added benefit was found for 29% of the 62 mAbs. For 22.6% of the drugs, a minor additional benefit could be proven and 17.7% showed no evidence of an additional benefit. Furthermore, no additional benefit could be quantified for 22.6% of the 62 drugs and no GBA assessment was available for 8.1% (Fig. 8).

### Development of the TOP 8

**Fig. 9:**
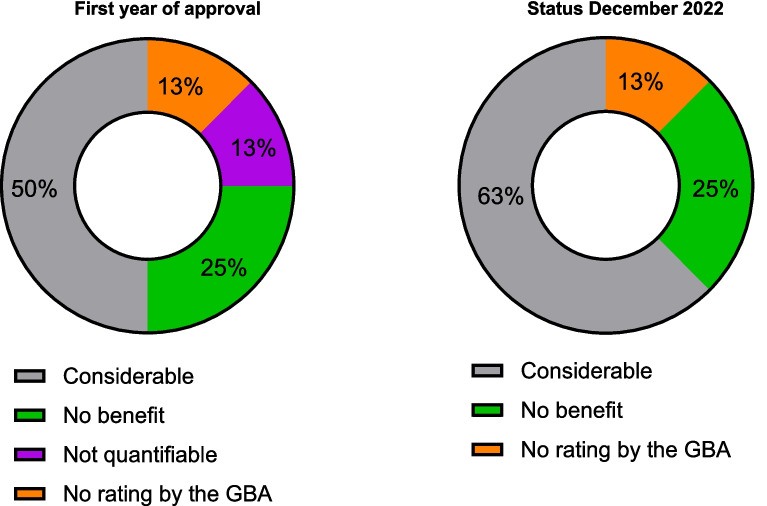
GBA benefit assessment of initial indication after initial approval and as of 12/2022. Values rounded; discrepancies due to rounding

In its additional benefit assessment, the JCC determines a considerable additional benefit for 50% of the TOP 8 drugs (Fig. 9). For 25% of the TOP 8, no additional benefit can be proven, for 13% of the TOP 8 the benefit is not quantifiable and another 13% had no GBA rating. In a (re)evaluation (as of 12/2022), Daratumumab was changed from a non-quantifiable added benefit to a considerable added benefit, which leads to an increase of 13% in the category of a considerable adiitional benefit. In the Fig. 9, no additional approved indication is considered.

**Fig. 10:**
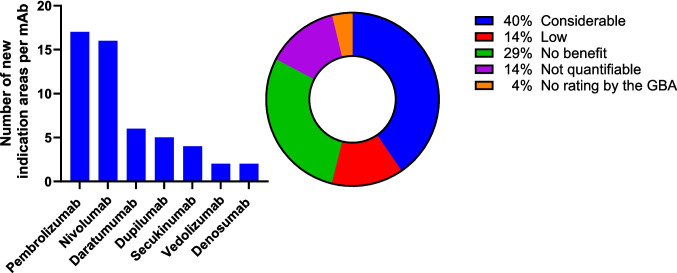
Left: Number of new indications per drug of the TOP 8; right: List of additional benefit in percent for the newly approved indication areas of the TOP 8

In the period from initial approval up to and including 2021, there are a total of 52 newly approved indications for the TOP 8. Pembrolizumab leads with 17 new indications, followed by Nivolumab with 16 new indications. The remaining six monoclonal antibodies follow at a considerable distance with two to six new indications each. Of the 52 new indications, 40.4% show a considerable additional benefit, 13.5% a minor additional benefit and no additional benefit can be proven for 28.8% (Fig. 10). An additional benefit cannot be quantified for 13.5% of the new indications in the TOP 8 and no GBA assessment is available for 3.8%.

A re-evaluation of the added benefit by the JCC was carried out for four of the 52 new indications, whereby the assessment deteriorated for two, improved for one new indication and came to the same result as in the initial assessment for another.

### Development of the FLOP 5

**Fig. 11:**
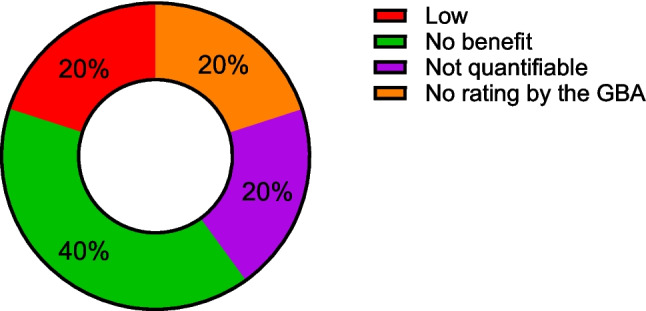
GBA assessment of the additional benefit of the FLOP 5

In the assessment of the added benefit by the JCC, a minor added benefit could be proven for 20% of the FLOP 5 and no added benefit for 40%. For 20% of the FLOP 5, an added benefit could not be quantified or no GBA assessment was available (Fig. 11). For none of the FLOP 5 was a further new indication applied for since the initial approval or a re-evaluation of the assessment carried out.

### MAbs without a proven additional benefit by the GBA

The proportion of drugs without proven added benefit is highest in the FLOP 5 group at 40%. In the overall analysis of the 62 monoclonal antibodies, the proportion of drugs without proven added benefit is lower (17.70%) than in the TOP 8 category (25%). In the re-evaluation of the added benefit 2021 for the TOP 8 and FLOP 5, there is no change in the proportions of drugs without evidence of added benefit. In a direct comparison of the TOP 8 without all therapeutic indications and the TOP 8 with all 52 therapeutic indications, the proportion without proven added benefit is higher in the group of TOP 8 with the 52 therapeutic indications at 28.8% (28.8% vs. 25%). After the (re)evaluation (as of 12/22), the proportion of the TOP 8 with all therapeutic indications rises to 30.8%.

### MAbs without quantified added benefit

In the TOP 8 group, 12.50% of the drugs have a non-quantifiable added benefit, which is the smallest proportion compared to the overall analysis from 2010 to 2021 and the FLOP 5. In the overall analysis, 22.60% have a non-quantifiable added benefit and in the FLOP 5 group the proportion is 20%. In the (re)evaluation as of 12/2022, there are no drugs in the TOP 8 group with a non-quantifiable added benefit. In the FLOP 5 group, the proportion of drugs without quantifiable added benefit remains at 20%. The proportion of medicinal products without quantifiable added benefit in the group of the TOP 8 with the entire 52 therapeutic indications is 13.5% and 7.7% after the (re)evaluation.

### MAbs without GBA assessment

In the FLOP 5 group, the proportion of drugs without a GBA assessment is 20%, which is also the largest proportion in contrast to the overall analysis and the TOP 8 group. In the overall analysis, this proportion is 8.10% and in the TOP 8 group 12.5%. In the re-evaluation, there is no change in the TOP 8 or FLOP 5 with regard to drugs without GBA assessment. The TOP 8 with all therapeutic indications have a share of 3.8% in the category of medicinal products without JCC assessment. After the (re)evaluation, the share increases to 5.8%.

### MAbs with a minor additional benefit

The proportion of drugs with a minor added benefit is highest in the overall analysis at 22.60%, while no drug with a minor added benefit is recorded in the TOP 8. In contrast, 20% of the FLOP 5 drugs correspond to a minor added benefit. Here, too, there is no change after the (re)evaluation. In the group of the TOP 8 with all therapeutic indications, the proportion of drugs with a minor additional benefit is 13.5%, which shows no change up to 12/2022.

### MAbs with considerable additional benefit

In the category of drugs with a considerable added benefit, the TOP 8 group has the largest share with 50%. In the overall analysis of the 62 monoclonal antibodies, the proportion of drugs with considerable added benefit is 29%. In the group of the FLOP 5, no drug has a considerable added benefit. According to the (re)evaluation, 62.50% of the drugs in the TOP 8 group have a considerable added benefit. The proportion of the TOP 8 with a total of 52 therapeutic indications in the category of drugs with considerable added benefit is 40.4%. This increases to 42.3% after (re)evaluation.

### Evaluation by the ESMO

**Fig. 12:**
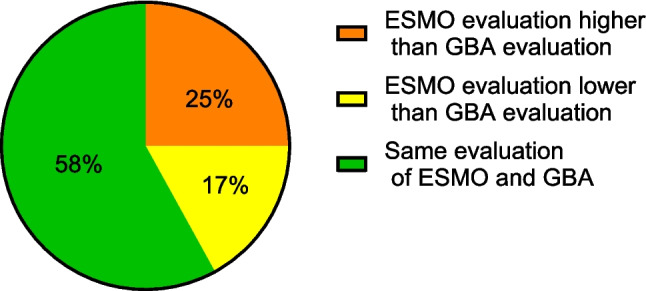
Similarities and differences in the evaluation of the monoclonal antibodies with an oncological indication by the GBA and ESMO (*n* = 12)

Of the 27 monoclonal antibodies with an oncology indication, 12 had an ESMO evaluation. When comparing the GBA evaluation and the ESMO-MCBS, 58% of the evaluations are the same, 25% of the ESMO evaluation feature a better evaluation than the initial GBA evaluation and 17% of the ESMO evaluations show a lower rating, than in the GBA evaluation (European Society for Medical Oncology, (https://www.esmo.org/guidelines/esmo-mcbs/about-the-esmo-mcbs, last accessed 06.06.2025)) (Fig. 12).

### Advertising

**Fig. 13:**
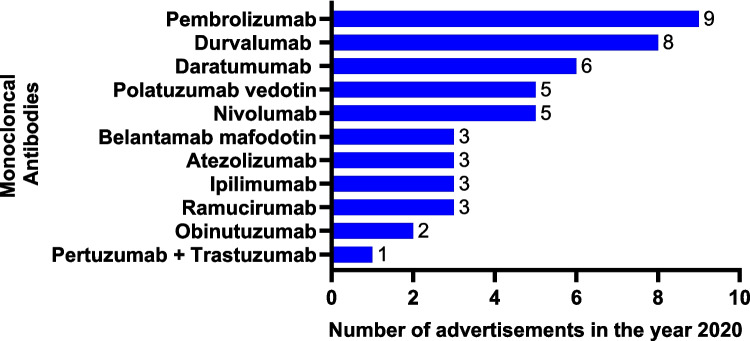
Listing of monoclonal antibodies according to the number of advertisements in the journal “Oncology Research and Treatment” 2020 (*n* = 10 issues)

In the analysis of the ten issues of the journal “Oncology Research and Treatment,” monoclonal antibodies are advertised a total of 48 times. Pembrolizumab is at the top of the list in terms of the number of advertisements with 9 (Fig. 13). It is closely followed by Durvalumab with 8 advertisements and Daratumumab with 6 advertisements. Seven other monoclonal antibodies and one monoclonal antibody combination were advertised in the ten issues, with between 1 and 5 ads. 27% of the 48 monoclonal antibodies advertised belong to the TOP 8 group, which account for 42% of the total number of monoclonal antibody advertisements (“Oncology Research and Treatment” 2020).

## Discussion

### Prescriptions and DDD

The increasing prescriptions and the rising number of DDDs of monoclonal antibodies show the increased demand as well as the growing relevance of new targeted agents. Due to the limited therapeutic range of cytostatic drugs and the resulting side effects, the development of new active substances that are more targeted is moving into focus (Ludwig et al. 2022).

### Net costs

The dramatic increase in the net cost of monoclonal antibodies in the overall pharmaceutical market, as well as their share among the top 30 best-selling drugs are comparatively low in contrast to the prescription figures. In 2021, 38% of the top 30 best-selling drugs were monoclonal antibodies. However, none of the monoclonal antibodies were listed among the 30 most frequently prescribed drugs. The direct comparison with other drug groups highlights the significant difference in the net cost-prescription ratio (Ludwig et al. 2022). Pembrolizumab from the TOP 8 category has the highest net costs. In this analysis, Pembrolizumab shows a clear incongruence between net costs and prescriptions. At €1,129.53 million, Pembrolizumab has the highest net costs of the 62 drugs analyzed and ranks first among the top 30 drugs by net costs. The DDD in 2021 is 4.26 million, but the prescriptions are only 405.8 thousand. Pembrolizumab thus far outperforms the other drugs being compared and all drugs on the market. This compares with Nivolumab, which is also a PD1 inhibitor and is approved for several of the same indications. Both have a considerable additional benefit and both are approved for several indications (Pembrolizumab 17 vs. Nivolumab 16 (as of December 31, 2021)). However, it is striking that the net costs of Pembrolizumab in 2021 are around €657 million higher than those of Nivolumab, but the prescription figures for Pembrolizumab are around 24.2 thousand lower. The described incongruity can be largly attributed to the diffrences in their dosing intervals. Pembrolizumab is typically administered every three weeks, wherareas Nivolumab follows a two-week schedule. Consequently, the higher number of presciptions observed for Nivolumab reflects the increased frequency of administrations per patient rather than a larger treated population (European Medicins Agency, 2025).

Despite the lower prescription counts for Pembrolizumab, its net costs are consistently higher, a discrepancy explained by the elevated annual treatment costs per patient. During the first year of registration, the annual treatment costs per patient for Pembrolizumab amounted to €144.056,64^1^ at a dose of 200 mg per treatment day, with a treatment interval of three weeks and including statutory deductions. In comparison, the annual treatment costs for Nivolumab amounted € 106.465,32€^2^ during the first year after registration, at a dose of 240 mg per treatment day and a treatment interval of two weeks. Interestingly, although Nivolumab is administered at a higher dose and more frequently than Pembrolizumab, the annual DDD for Pembrolizumab can surpass that of Nivolumab. This result arises because the Defined Daily Dose (DDD) is a standardized, non-therapeutic unit that does not accurately capture true drug exposure, treatment duration, or financial impact (“DDD schnell erklärt,” 2025; Fuchs et al 2012; Wasem and Bramlage 2008).

Collectively, these considerations account for the observation, why Pembrolizumab demonstrates both higher net costs and higher DDDs, despite having lower prescription counts than Nivolumab.

It remains unclear why the annual treatment costs associated with Pembrolizumab substantially exceed those of Nivolumab, despite their comparable mechanisms of action. Especially when current retrospective studies such as Moser et al. from 2020 or Torasawa et al. from 2022 show no significant difference between Nivolumab and Pembrolizumab in the indications NSCLC or advanced melanoma. The studies relate to the endpoints of overall survival and safety (Moser et al. 2020; Torasawa et al. 2022).This leaves the question of what justifies this large price difference unanswered.

With regard to the FLOP 5 (Necitumumab, Idarucizumab, Gemtuzumab Ozogamicin, Bezlotoxumab, Ibalizumab, possible reasons for the low net costs and prescription figures may be the GBA assessments, which are predominantly declared as unproven, unquantifiable or low in the FLOP 5 category. The low prescriptions and net costs in the case of Necitumumab can be explained by the fact that it is no longer marketable. Necitumumab was withdrawn from the market in February 2021 as the pharmaceutical company did not apply for an extension of the marketing authorization (European Medicines Agency 2021).

### Assessment of the added benefit by the JCC (*n* = 62)

In this analysis, the highest assessment, which is defined as a significant added benefit, was not awarded. The JCC assessed a total of 62 monoclonal antibodies with a considerable, minor, non-quantifiable or no evidence of added benefit.

### MAbs without GBA evaluation (*n* = 62)

Some monoclonal antibodies, such as Denosumab, do not have a G-BA assessment, as they were approved before the Act on the Restructuring of the Pharmaceutical Market (AMNOG) of January 1, 2011. Since the Act, the prices for new patent-protected medicinal products have been set on the basis of an additional benefit assessment carried out by the Federal Joint Committee. In the case of Denosumab, a subsequent procedure was discontinued.

### MAbs without evidence of additional benefit (*n* = 62)

The proportion of monoclonal antibodies without proven added benefit by the JCC is higher in the FLOP 5 category than in the overall analysis of the 62 monoclonal antibodies as well as in the TOP 8 category. This applies both at the time of first authorization and at the time of (re)evaluation. This distribution illustrates that it can be assumed that the drugs with the lowest prescription figures, DDDs and net costs also tend to have a poorer added benefit assessment than the drugs with particularly high prescription figures, DDDs and net costs.

### MAbs without quantifiable additional benefit (*n* = 62)

An assessment without quantifiable additional benefit applies in this analysis to more than one fifth of the 62 drugs (22.60%). Likewise, in the assessment of the TOP 8 with a total of 52 indications, no added benefit can be quantified for 13.50%, although these drugs belong to the TOP 8 in the categories net costs, prescriptions and DDD. This incongruence raises the question of how drugs without a quantified added benefit can have such high prescription figures and net costs.

### MAbs with little additional benefit (*n* = 62)

The proportion of drugs with a minor added benefit is highest in the overall analysis of the 62 monoclonal antibodies at 22.60%. It is striking that the FLOP 5 have a higher proportion of drugs with minor added benefit than the TOP 8 or the TOP 8 with the 52 indication areas. This applies both at the time of initial approval and after the (re)evaluation in 2021. This result is surprising, as a better GBA evaluation is usually associated with higher prescriptions and net costs.

### MAbs with considerable additional benefits (*n* = 62)

The largest proportion of drugs with a considerable added benefit falls into the TOP 8 category, and the largest proportion of drugs in the TOP 8 category with 52 new indications and the overall analysis also have a considerable added benefit. In the FLOP 5 category, there are no drugs with significant added benefit. This illustrates that there is a certain congruence between the drugs with the highest net costs, prescription numbers and DDDs and a positive added benefit assessment. However, it is important to emphasize that in the category of the TOP 8 with 52 new indications and also in the overall analysis, although the largest proportion of drugs show a considerable added benefit, this is below 50% in both categories. Of the TOP 8 with 52 new indications, just 40.40% show a considerable additional benefit. Likewise, in the overall analysis, only 29% of the drugs show a considerable additional benefit. One would expect the drugs with the highest sales and prescription volumes in particular to have a predominantly considerable additional benefit that would justify the high costs.

### Overall assessment by the JCC

In the overall analysis of the 62 monoclonal antibodies, no overwhelming considerable added benefit can be proven (29%). Instead, almost one-fifth (17.70%) show no evidence of additional benefit and for slightly more than one-fifth (22.60%) no additional benefit can be quantified. Nevertheless, the number of newly approved monoclonal antibodies is increasing, as are the costs and prescriptions of the individual drugs. However, in the TOP 8 and FLOP 5 categories there is congruence between the net costs, prescriptions and DDDs and the assessed added benefit.

### Indication areas

Oncologicals are by far the main indication area for monoclonal antibodies. It is therefore not surprising that monoclonal antibodies account for the highest costs in the oncology indication group in 2021 at €4.3 billion. As the costs of monoclonal antibodies rise, the costs of oncologicals also rise, so that in 2021, as in previous years, this indication group is by far the indication group with the highest turnover in the SHI pharmaceutical market (Ludwig et al. 2022). But what is surprising, is that these monocolonal antibodies have high costs despite the low prescription figures. In 2021 monoclonal antibodies accounted for only 0.35% of the total pharmaceutical market prescriptions, yet they accounted for 8.60% of the total pharmaceutical market sales. One reason for this divergence is that the drug prize is set by the pharmaceutical company within the first year and only after that year the negotiated reimbursement will take in effect (Gemeinsamer Bundesausschuss 2009). This in addition to the generally high costs of monoclonal antibodies, leads to even higher total pharmaceutical prices, which can not be stustained by the health care system in the long term.

### Further additional benefit assessments

The contrasting additional benefit assessments from the G-BA and ESMO demonstrate that distinct methodological approaches can yield varying outcomes. The ESMO-MCBS incorporates both overall survival and additional factors, including progression-free survival (PFS), disease free survival (DFS), quality of life (QOL) and the prognosis of the condition and toxicity, whereas the GBA primarily considers endpoints from the categories of mortality, morbidity, and health-related quality of life. Therefore, a study endpoint such as PFS can be assigned a high rating by ESMO-MCBS if it signifies a substantial improvement. However, the GBA may categorize the same endpoint as being inadequately patient-relevant and not classifying this effect as an added benefit. Consequently, the same data may result in a lower assessment of added benefit by the GBA, while ESMO-MCBS provides a better assessment (Cherny et al. 2025; European Society for Medical Oncology n.d.; Ruof et al. 2014). This discrepancy is evident in the analysis, which reveals that of the 12 monoclonal antibodies with oncological indications and ESMO ratings, 58% correspond to the GBA rating. However, 25% of the monoclonal antibodies received a higher ESMO rating than the GBA would have assigned. The disparity makes it difficult to make an evidence-based decision regarding the choice of therapy (Obst and Seifert 2023).

Furthermore differing assessments make it difficult to assess the benefits of a drug. Not only does it make  it difficult to justify the ever-increasing costs of drugs, especially monoclonal antibodies, which the GBA often considers to be of no additional benefit, but it also leads to contradictions between GBA evaluations and official guidelines. A study in 2017 conducted for the Association of Research-Based Pharmaceutical Companies (vfa) revealed, that GBA-assessments and guidelines often did not align, because of the discordance of a patient-specific situation as opposed to a rigid treatment algorithm (Laschet 2017). This explains high prescription rates and net costs, even for drugs that do not offer significant additional benefits. It also highlights the need of improved cooperation between the GBA and other professional institutions.

### Advertisements

The advertisements of monoclonal antibodies in the 10 issues from the year 2020 of the journal “Oncology Research and Treatment” advertise 71% of targeted drug therapies such as monoclonal antibodies and protein kinase inhibitors (Obst and Seifert 2023). Three of the twelve advertised mAbs, i.e. Pembrolizumab, Daratumumab and Nivolumab are among the TOP 8 in terms of net costs, prescriptions and DDD. Particularly striking is Pembrolizumab which, with 9 advertisements, has the highest number of advertisements and is also the top-selling drug. This shows a correlation between sales and the number of advertisements.

### The case of Pembrolizumab

Pembrolizumab (Keytruda) is a PD-1 receptor antibody that was launched on the German market in August 2015 for the indication of advanced melanoma. It is the top-selling oncology drug in 2021 with net costs of € 1129.5 million, an increase in costs of 29% and an increase in prescriptions of 26.8% within one year (2020 vs. 2021). The JCC assessed Pembrolizumab as having a considerable additional benefit when it was launched. Since the new launch, 16 additional indications for Pembrolizumab have been approved (as of December 2021), of which 58.8% have a considerable added benefit, 5.9% each have a minor or non-quantifiable added benefit and 29.4% have no added benefit (JCC). Pembrolizumab therefore has a considerable added benefit for just over half of the indications. In contrast, Pembrolizumab also shows no added benefit for more than a quarter of the indications. This represents a stark contrast to the high net costs and prescription figures and is a further example of the incongruence between the added benefit assessment and prescription figures and costs.

The high prescription figures and net costs can be explained on the one hand by the increasing number of approved indications, and on the other hand Pembrolizumab also has the highest number of advertisements in the oncology journal “Oncology Research and Treatment,” which may also be related to increased prescription figures and net costs.

### The case of Vedolizumab

Vedolizumab is an anti-${\alpha }_{4}{\beta }_{7}$ integrin antibody used in adult patients with moderate to severe Crohn’s disease and ulcerative colitis who have had an inadequate response to conventional therapy or one of the TNF-α inhibitors. Vedolizumab is among the TOP 8 in this analysis with net costs of €298.8 million, 117.8 thousand prescriptions and DDD of 7.9 million. In the case of Vedolizumab, the lack of congruence between net costs, prescriptions and DDD can be demonstrated with regard to the added benefit, as Vedolizumab does not show any demonstrable added benefit in the JCC assessment, although it is among the TOP 8 and is ranked 20th among the top 30 drugs by net costs in 2021. The GBA justifies the assessment with the lack of direct comparative studies compared to the appropriate comparator therapy (Gemeinsamer Bundesausschuss 2009). The Drug Commission of the German Medical Association (AKDÄ) also mentions the significantly higher clinical response rate compared to placebo, but also emphasizes the lack of direct comparisons with other drugs for the treatment of inflammatory bowel diseases. Furthermore, the AKDÄ assessment points out the low clinical remission rate, as well as serious side effects and the high costs (Arzneimittelkommission der deutschen Ärzteschaft 2015). In contrast to the assessment of the AkdÄ and the GBA, the DGVS and the DGIM assess a considerable additional benefit compared to Adalimumab in anti-TNF-α antibody-naïve patients with Crohn’s disease and ulcerative colitis in a statement of the GBA assessment of Vedolizumab, and in anti-TNF-α -antibody failures “Gemeinsame Stellungnahme der Deutschen Gesellschaft für Gastroenterologie 2015).

## Limitations of this study

The prescription figures, net costs and DDD in this study only refer to mAbs that were prescribed on an outpatient basis and only to those insured by the statutory health insurance and only dispensed via public pharmacies. Medicines prescribed via private health insurance or in hospitals are excluded. This study only considers monoclonal antibodies that were newly launched on the German market from 2010 up to and including 2021. This means that newly approved indications from 2022 onwards are not included in the 62 mAbs analyzed.

## Conclusion and outlook

This analysis shows that monoclonal antibodies are becoming increasingly important in the treatment of many different indications. Especially well-represented are monoclonal antibodies for treatment of malignant diseases. It is conspicuous, that according to the GBA, no significant additional benefits were assigned for most of the analyzed monoclonal antibodies. Particularly after initial approval, new indications are frequently developed that often do not provide an additional benefit as well. In this case only 40% of the 52 new indications of the TOP 8 offer significant added benefit, which does not justify the high prices.

Reassessments by the JCC, for example in the event of new data or after the deadline has expired, can both improve and worsen the initial assessments. A precise assessment and reassessment of these drugs is essential, particularly due to rising drug costs for newly approved monoclonal antibodies. A difficulty is the disparity in different benefit assessments. This makes an evidence-based decision regarding a choice of therapy challenging. Therefore, an improved cooperation between the GBA and other professional institutions is needed.

The high costs of new drugs is not only a well-known phenomenon in Germany, but is also perceived as a burden for healthcare systems and patients in other countries (Ludwig et al. 2022). The high prices of new drugs are often justified by the high costs of research and development. However, several publications have shown that the costs for research and development are significantly lower than often stated by the pharmaceutical company (Ludwig et al. 2022; Vokinger et al. 2021). Another justification for the high prices of monoclonal antibodies would be the superiority over standard compound-based pharmacotherapy. This promts the question, whether monoclonal antibodies are really superior to standard compound-based pharmacotherapy. There is no simple or general answer for that question. Monoclonal antibodies belong to the targeted therapy, which are known for their high specificity towards antigens, which offers the possibility of a personalized treatment according to the type of cancer or antigen expressed. Furthermore an advantage are the fewer adverse effects than conventional therapies, as well as the resistance to pathogen mutations, extended half-lives and versatile administration options (Damián-Blanco et al. 2023; Singh et al. 2023; Su et al. 2023).

These advantages depend on the kind of malignant disease or underlying pathological mechanism. Because of the individual effect, it is precisely why a structured and detailed benefit assessment, as well as regular reassessments over time, is essential, especially when the therapies account for such a large proportion of the costs of statutory health insurance and simultaneously in this case according to the GBA do not provide an overall significant benefit assessment. The high costs, together with demographic change, can become a burden. A burden that often cannot be justified by the benefits.

There is, however, a certain correlation between the mAbs with the lowest DDDs, prescription figures and net costs and the GBA’s assessment. This indicates, that the GBA-rating exerts some degree of influence, what still needs to be expanded upon.

With the SHI Financial Stabilization Act of October 2022, this is to be better implemented by negotiating higher discounts with companies for drugs without proven or minor additional benefits. Furthermore, the negotiated reimbursement price for new medicines is to be claimed from the seventh month and not only after one year. The sales threshold for drugs for the treatment of rare diseases, so-called orphan drugs, is also to be lowered from 50 to 30 million euros (Bundestag 2022). The future of this matter is yet to be determined, whether the new law can ensure the financial stability of statutory health insurance in Germany.

## Data Availability

All source data for this study are available upon reasonable request from the authors.

## Abbreviations

AkdÄ: Drug Commission of the German Medical Association (Arzneimittelkomission der deutschen Ärzteschaft)
ALL: Acute lymphoblastic leukemia
AML: Acute myeloid leukemia
ASZT: Autologous stem cell transplantation
AVR: Drug prescription report (Arzneiverordnungsreport)
CLL: Chronic lymphocytic leukemia
DFG: German research foundation (Deutsche Forschungsgemeinschaft)
DLBCL: Diffuse large B-cell lymphoma
DDD: Defined daily dose
DGIM: German society for internal medicine (Deutsche Gesellschaft für Innere Medizin)
DGVS: German society for gastroenterology, digestive and metabolic diseases (Deutsche Gesellschaft für Gastroenterologie, Verdauungs- und Stoffwechselkrankheiten)
dMMR: Mismatch repair deficiency (deficient mismatch repair)
DRG: Diagnosis-related-groups
DSF: Disease-free survival
EGFR: Epidermal growth factor receptor
EMA: European Medicines Agency
GBA: Joint Federal Committee (Gemeinsamer Bundesausschuss)
GKV: Statutory health insurance (SHI) (Gesetzliche Krankenversicherung)
HER2: Human epidermal growth factor receptor 2
InEK: Institute for the hospital fee system (Institut für das Entgeldsystem um Krankenhaus)
MAb: Monoclonal antibodies
MSI-h: High-grade microsatellite instability
NSCLC: Non-small cell lung cancer
QoL: Quality of life
SGB: German Social Code (Sozialgesetzbuch)
PFS: Progression-free survival
SLE: Systemic lupus erythematosus
VAT: Value added tax
VFA: The research-based pharmaceutical companies (Verband der forschenden Pharma-Unternehmen)
WIdO: Scientific Institute of the Local Health Insurance Funds (AOK) (Wissenschaftliches Institut der AOK)

## Footnotes

1 Lauer-Taxe Stand 01.01.2016.

2 Lauer-Taxe Stand 01.01.2016.
